# Uptake of COVID-19 Vaccination in a Medium Secure Psychiatric Hospital Population

**DOI:** 10.1192/bjo.2022.217

**Published:** 2022-06-20

**Authors:** Simon Gibbon, Emma McPhail, Georgina Mills, Martin McBride, Rebakah Storer, Nicholas Taylor, Lucy McCarthy

**Affiliations:** Nottinghamshire Healthcare NHS Foundation Trust, Nottingham, United Kingdom

## Abstract

**Aims:**

Compared with the general population, people with mental health disorders are at increased risk of negative physical and mental health outcomes following SARS-CoV-2 infection. In the UK, all adult mental health in-patients were offered COVID-19 vaccination as a priority group. Patients admitted to medium secure care have greatly increased mortality compared with the general population. Understanding COVID-19 vaccine uptake, and reasons for refusal, in patients in medium secure hospitals is important given the high prevalence of chronic physical health comorbidities such as obesity and diabetes, as these conditions are also associated with poor clinical outcomes in COVID-19 disease. Aims: To assess the proportions of patients who accepted or declined the COVID-19 vaccine, and explore their reasoning. To examine vaccine uptake between White and Black Asian minority ethnic (BAME) patients, and between younger/older patients.

**Methods:**

The study took place at a medium secure hospital with male and female inpatients. All patients were offered a COVID-19 vaccine, and had a capacity and physical health evaluation completed by their Consultant Forensic Psychiatrist.

**Results:**

Data regarding capacity to consent to the vaccine, acceptance/refusal, and demographics were retrospectively collected from the clinical records. In total, 85 patients (92.4% of eligible patients) had capacity to decide if they wanted the COVID-19 vaccine. Of these 68 (80.0%) consented and 17 (20.0%) declined to consent.

A similar proportion of patients aged under and over 40 years old consented. Those from a BAME background were more likely to decline than White British patients. The reasons for capacitous refusal appeared similar to the general population.

**Conclusion:**

COVID immunisation was broadly acceptable for patients in medium secure hospitals. The prevalence and reasoning of capacitous refusal appears similar to the general English population.

The indication that BAME patients were more likely than White patients to decline the vaccination echoes the findings of research conducted in the Leicester general hospital. Further consideration needs to be given to how the uptake of COVID-19 vaccination can be improved in people with BAME ethnicity, especially as this group is also overrepresented in secure hospital settings.

The study demonstrates that similar services should be able to approach the vaccination process with confidence. As many people with severe mental disorder also have high physical comorbidity that would increase the risk of a poor clinical outcome if they contracted COVID-19, protecting this vulnerable population through vaccination must be a priority for mental health services.

